# Linkages between animal and human health sentinel data

**DOI:** 10.1186/1746-6148-5-15

**Published:** 2009-04-23

**Authors:** Matthew Scotch, Lynda Odofin, Peter Rabinowitz

**Affiliations:** 1 Yale Center for Medical Informatics, Yale School of Medicine, Yale University, New Haven, CT USA; 2 Department of Epidemiology of Microbial Diseases, Yale School of Public Health, Yale University, New Haven, CT USA; 3 Yale Occupational and Environmental Medicine Program, Yale School of Medicine, Yale University, New Haven, CT USA

## Abstract

**Introduction:**

In order to identify priorities for building integrated surveillance systems that effectively model and predict human risk of zoonotic diseases, there is a need for improved understanding of the practical options for linking surveillance data of animals and humans. We conducted an analysis of the literature and characterized the linkage between animal and human health data. We discuss the findings in relation to zoonotic surveillance and the linkage of human and animal data.

**Methods:**

The Canary Database, an online bibliographic database of animal-sentinel studies was searched and articles were classified according to four linkage categories.

**Results:**

465 studies were identified and assigned to linkage categories involving: descriptive, analytic, molecular, or no human outcomes of human and animal health. Descriptive linkage was the most common, whereby both animal and human health outcomes were presented, but without quantitative linkage between the two. Rarely, analytic linkage was utilized in which animal data was used to quantitatively predict human risk. The other two categories included molecular linkage, and no human outcomes, which present health outcomes in animals but not humans.

**Discussion:**

We found limited use of animal data to quantitatively predict human risk and listed the methods from the literature that performed analytic linkage. The lack of analytic linkage in the literature might not be solely related to technological barriers including access to electronic database, statistical software packages, and Geographical Information System (GIS). Rather, the problem might be from a lack of understanding by researchers of the importance of animal data as a 'sentinel' for human health. Researchers performing zoonotic surveillance should be aware of the value of animal-sentinel approaches for predicting human risk and consider analytic methods for linking animal and human data. Qualitative work needs to be done in order to examine researchers' decisions in linkage strategies between animal and human data.

## Introduction

In recent years, there has been increasing awareness on the part of both human and animal health professionals that disease events in animal populations may have direct relevance to human health. As with the analogy of the "canary in the coal mine", animals may serve as sentinels of human health threats in the environment, and work by Halliday and others [1] have focused on establishing a framework to facilitate surveillance efforts. Examples of sentinels include the emergence of zoonotic diseases in wildlife populations concurrent with a novel outbreak of disease in humans such as West Nile Virus (WNV) [2,3], SARS [4,5], and Avian Influenza [6,7]. As a result of these recent events, there has been a heightened emphasis on the use of surveillance efforts in both domestic and wild animal populations. This includes the worldwide surveillance of wild birds for avian influenza. In 2006, the United States Geological Survey, sampled more than 193,000 birds in the US alone as part of their Wild Bird Surveillance Plan [8]. On a global scale, The Global Avian Network for Surveillance (GAINS) surveillance system, funded by US AID, has one of the leading avian surveillance systems and has over 100,000 birds included in their electronic database [9].

This awareness of the shared risk faced by animal and human populations has led to a call for a "One Medicine" approach [10] (now called "One Health"), of communication and interdisciplinary practice between veterinary and human medical professionals. A key part of such an approach is "Joint cross-species disease surveillance and control efforts in public health" (Kahn, L., B. Kaplan, and T.P. Monath: One Health Mission Statement, unpublished). There have been a number of attempts to link human and animal health data such as the tracking of syndromic events in pet animals [11] or the collection of data on sentinel birds for West Nile infection control [12-21]. Controversy exists about the value of such approaches. For example while Eidson et al reported that dead crow clusters predicted human WNV risk [22], Brownstein et al have reported that dead crow sightings are less reliable than mosquito surveillance for prediction of human WNV risk [15]. Similarly, no clear correlations have been published to date from ongoing surveillance of pet populations. Perhaps the clearest example of routine use of animal disease data is in national and state rabies surveillance, in which 49 states and Puerto Rico participated in the monitoring and reporting of nearly 7,000 animal cases (and 3 human cases) to the CDC [23].

Animal health surveillance efforts and human health surveillance efforts are often separate initiatives resulting in the data being stored in separate and potentially vastly different databases. Careful attention and planning must be done if these data sources are to be effectively linked. There is a belief that automated systems to integrate public health data can enhance the surveillance process. The design and development of these systems requires experts in public health informatics, which is the study of the acquisition, storage, and management of electronic public health data for identifying and controlling health issues in the population [24]. Much of the public health informatics work has been in the development of biosurveillance systems that automatically merge disparate health, environmental, geographical, consumer, and population data to model and detect aberrations that might signify a public health priority. Examples include the Real-Time Outbreak and Disease Surveillance Systems (RODS) [25] and the Automated Epidemiologic Geotemporal Integrated Surveillance Systems (AEGIS) [26] that use electronic hospital syndromic information to predict bioterrorism and naturally occurring outbreaks such as influenza.

In order to identify priorities for building integrated surveillance systems that effectively model and predict human risk of zoonotic disease, there is a need for improved understanding of the practical options for linking surveillance data of animals and humans. For this study, we performed an analysis of the Canary Database [27], an online resource for literature on the animal-sentinel interface, in order to examine the use of how animal and human data is linked for analysis purposes. The Canary Database does not represent the entire spectrum of epidemiological papers of animal-human events. However, the purpose of this paper is not an exhaustive search of the literature on animal-human health events. Rather, the intent is to highlight the use of various linkage strategies for animal and human data and discuss implications of these strategies for public health research. In addition, both humans and non-human animals share the same ecosystem and while it is possible that animals can serve as sentinels for humans, there are times when a sentinel event in humans has implications for animal health. The Canary Database, however, is oriented toward animals as sentinels for human health hazards, therefore for the remainder of this paper, we use the term 'animal sentinel' to mean an instance in which a non-human animal might indicate concurrent or future health risk to humans. However, we believe that our findings regarding linkage between human and animal data streams have relevance to both human health and animal health.

## Methods

The Canary Database [27] is a publicly accessible online database of studies in the biomedical literature concerned with animals as sentinels of zoonotic, environmental, and toxic effects on human health. Curators of this database include both human and animal health professionals who periodically search the medical bibliographic databases such as PubMed and identify, using predetermined algorithms, studies that analyze the effects of zoonotic and environmental health hazards on free ranging animal populations, including companion, livestock, and wildlife animals. Articles are curated into the database according to study methodology, hazards and outcomes studied, animal species, and location. Currently, there are over 1,600 articles in the database going back to 1966.

Using the advanced search feature in the Canary Database, an initial group of papers were retrieved that described at least a shared health outcome between animals and humans or a shared exposure (e.g. of an environmental toxin). Then, two of the reviewers (MS and LO) reviewed the curated information for each of these papers and if needed, the papers themselves, to determine if they were original investigations. Review articles and media analysis (newspaper or magazine articles) were not included. In addition, papers describing laboratory-based toxicology work were not included.

For each paper in the study, we defined their linkage category, based on consensus among the authors, to be at least one of four possibilities:

◦ *Analytic*: Studies that use quantitative methods to assess human risk. For example, studies that use cases of WNV found in dead crows to quantify the risk of the disease in humans, would fall under this category.

◦ *Descriptive*: Studies that describe the health outcomes in both animals and humans, but do not attempt a quantitative linkage between the two. For example, a seroprevalence study that lists the number of positive Hantavirus cases in both rodents and humans in a particular area would fall under this category.

◦ *Molecular*: Studies in which the investigators use molecular epidemiological techniques to show similarities between strains of a pathogen occurring in animals compared to humans, thereby providing evidence as to whether or not species crossover of infection has occurred. For example, studies that use phylogenetic algorithms to assess the lineage of H5N1 Avian Influenza strains isolated in animals and humans would fall under this category.

◦ *No human outcomes*. Studies in which no outcomes in humans are considered. These studies often provide evidence of exposure in humans, but do not discuss outcomes related to human health. For example, a study that assesses the bioaccumulation of mercury in tilapia would fall under this category. Humans are considered exposed since they could eat the fish or drink the contaminated water but outcomes are not assessed.

Thus, the first three categories represent original investigations that describe health outcomes in both animals and humans, while the final category describes all papers that include only animal outcomes or no health outcomes at all.

Each paper was assigned to at least one of these four linkage categories, with some papers counted in two categories. For example a paper that describes the seroprevalence of a field study of Nipah Virus in fruit bats and humans and then develops a phylogenetic tree to assess the similarities would be considered both a descriptive linkage and a molecular linkage.

## Results

At the time of this study, Canary contained 1,661 articles from 1966 to 2007. Of these, 465 (28%) were judged to be appropriate original epidemiological investigations that related animal disease data to human disease data. These 465 papers were further analyzed.

Table 1 shows that the majority of studies linked animal and human data in a descriptive fashion (57%), thus describing or listing the number of cases in animals and humans without an attempt to predict future risk based on the results. This often includes the trapping of suspected animal reservoirs that are located in the vicinity of confirmed or suspected human cases. In these types of studies, results are often reported as percentage of positive cases in animals and percentage of positive cases in humans. Complicated or in-depth statistical analysis is often not conducted in descriptive linkage studies. Papers that used analytic methods ([2,13,15,18,20,21,28-50]) to link animal and human data consisted of 6% of the articles retrieved and the methods used in each are summarized in Table 2. For each paper, the primary author, the zoonotic disease, the animals studied (domestic, livestock, or wildlife), and our summary of the methods for analytic linkage are presented.

**Table 1:** Papers in our Canary search by type of Linkage Category.

**Linkage Category**	**Percent**
Descriptive	57%
No human outcomes	23%
Molecular	17%
Analytic	6%

A paper could be classified into multiple linkage categories.

**Table 2:** Papers in our Canary search using Analytic Linkage for human and animal health data

**First Author**	**Zoonotic Disease**	**Animals Studied**	**Description of Linkage**
Ferguson [32]	BSE	Livestock	Statistical modeling of human risk from transmission of infected sheep based on the number of animals slaughtered for food production
Keeling [35]	Plague	Wildlife	Stochastic metapopulation modelling using rodent population estimates to predict disease outbreaks in rats and subsequent severity of human infection
Niklasson [43]	Many	Wildlife	Cross-correlation function to determine dependence between abundance of bank voles and various diseases (and vice-versa) from hospitalization and cause of death registry.
Niklasson [42]	Puumala	Wildlife	Cross-correlation function to determine dependence between rodent density and human cases.
Eidson [20]	West Nile Virus	Wildlife	Number of dead crows sightings per square mile plotted against number of human cases
Dubey [30]	Toxoplasma Gondi	Companion, Livestock, and Wildlife	Logistic regression of cross-sectional data to examine risk of human infection for farm handlers with factors such as seroprevalence of cats, age of workers, and years of work
Gurtler [33]	Chagas Disease	Companion and Wildlife	Expected relative risk of infected bugs based on the combined effect of the presence of infected dogs and infected humans.
Rab [44]	Leishmaniasis	Companion	Association of human and canine disease risk shown through contingency table of households with and without infected dogs versus infected humans in a random sample
Zeman [49]	Lyme Disease	Wildlife	Correlation between population densities of game animals on GIS raster maps with human risk maps using a covariance/correlation matrix
Watson [2]	West Nile Virus	Wildlife	Relationship of Geocoded human cases and estimated dead crow densities used to calculate Mantel-Haenszel incidence ratio with Greenland- Robins 95%CI.
Mostashari [41]	West Nile Virus	Wildlife	Spatial-temporal cluster analysis of dead bird findings for early warning of known mosquitoes and human infection to produce risk maps.
Mitra [40]	Sarcoptes scabiei	Companion and Livestock	Association between infected male and female adults and children with infected sheep, goats, cattle and dogs, by using a contingency table
Marrie [39]	Q Fever	Companion	Risk factor for disease between cases and controls including exposure to stillborn kittens. Exposure to parturient cats, and slaughtering of animals assessed through chi-square, Fisher's, and Logistic regression
Wall [47]	Salmonellosis	Livestock	Logistic regression to analyze risk factors with infection including cross-sectional data such as contact with sick farm animals
Xu [48]	Hemorrhagic Fever	Wildlife	Risk of cat ownership and presence of rodent activity for infected versus non-infected calculated using Mantel-Haenszel
Brownstein JS [29]	West Nile Virus	Wildlife	Spatial cluster analysis of human cases and logistic regression according to normalized difference vegetation index (NDVI) were used to create disease risk maps which was validated using locations of virus-positive mosquitoes
Theophilides [46]	West Nile Virus	Wildlife	Risk model developed by Knox test using space and time data from dead bird reports and calibrated using human case reports
Shaman [45]	St. Louis Encephalitis	Livestock	Logistic regression model to predict epidemic using water table depth, as well as avian host virus susceptibility and mobility
Brownstein [15]	West Nile Virus	Wildlife	Regression Model including virus-positive mosquitoes and birds to predict human cases.
Andreadis TG [13]	West Nile Virus	Wildlife	Linear regression models to determine association between mosquito infection rates and number of human cases, and between the human population density and mosquito abundance.
Julian [34]	West Nile Virus	Wildlife	Association of early season crow activity including reported dead crows and infected crows with human infection, assessed through relative risk calculation for univariate analysis and logistic regression for multivariate analysis
Guptill [21]	West Nile Virus	Wildlife	Relative risk to determine association between counties reporting early season crow deaths and subsequent reporting of human infection
Zinsstag [50]	Brucellosis	Livestock	Deterministic model to estimate disease transmission from livestock to humans using demographic and seroprevalence data from cattle and sheep and human case data.
Mannelli [38]	Mediterranean spotted fever	Companion	Association between disease infection in canines and risk of disease in humans by performing second order neighborhood analysis on distance of dog residences from known human cases
Mannelli [37]	Lyme Disease	Companion	Risk for human exposure of disease vectors was calculated by relative risk using questionnaire data including past exposure to ticks and occupation
Li [36]	Many	Livestock	Probability model to estimate risk of human infection from exposure to vectors using estimates including number of vector bites per host per season and vector infection rate
Ezenwa [31]	West Nile Virus	Wildlife	Association between bird density and virus infection rates in mosquitoes and humans by simple linear regression and multivariate regression
Corrigan [18]	West Nile Virus	Livestock	Spatial scan statistic using Poisson distribution to predict clusters of infected horses and humans by combining cases in humans, horses, population data, location of the horses and location of the patient
Ascione [28]	None (Domestic Violence)	Companion	Stepwise Logistic regression to examine risk factors associated with physical abuse and threats of abuse to family pets including interview variables with abused and non-abused women such as physical spousal abuse, verbal abuse, and women's education level

### Analytic Linkage of Human and Animal Data Using Regression

Popular linkage approaches included multiple regression (Logistic, Linear, Poisson), spatial analysis (Cluster analysis, Digital mapping, etc.) and odds ratio/relative risk calculation. The purpose of these papers tended to use infection, death, or contact with animals as the main independent variable to predict risk of infection in humans. Many also used additional input variables such as interview and survey data to identify the most parsimonious model. For example a paper by Dubey et al [30] studying risk of Toxoplasmosis on US farms collected interview data from farmers and collected blood and fecal specimens from animals and farmers as well as soil and water samples. In order to study the association between the seroprevalence of farmers, the authors combined the various animal specimen data with farmer interview data in a logistic regression model. Seroprevalence of toxoplasmosis in cats was the only variable found to be significant. In another study, Brownstein et al [15] used a regression model to study the value of non-human surveillance programs on predicting risk in humans. The authors used data from the ArboNet system such as number of virus-positive birds, mosquitoes and humans and evaluated the models predictive ability by assessing the coefficient of determination (R^2^) [15].

### Analytic Linkage of Human and Animal Data Using Spatial Analysis

Many of the articles used spatial analysis in which georeferenced locations of infected cases or reported animal deaths were considered. This demonstrates the growing interest by epidemiologists and animal health researchers in examining spatial factors in relation to animal and human health monitoring. For example, the spatial scan statistic, developed by Kulldorff [51], has been widely used to identify disease clusters. The statistic scans temporal or spatial data, calculates the number of observed and expected observations (infections, deaths, etc.) using a shape (for spatial analysis) such as a circle or ellipse or an interval (for temporal analysis). The method then uses the likelihood ratio test to determine if the area inside the shape is significantly different than the outside area [51,52]. Statistical significance of the cluster with the maximum likelihood is then assessed by Monte Carlo simulation testing. Many of the analytic linkage papers used spatial scan functions to identify clusters of events such as animal deaths or infected cases of humans or animals. For example, Brownstein et al. [29] studied vegetation index data as a means to identify clusters of West Nile Virus in mosquitoes in New York City in 1999 [29]. The authors obtained human case information for New York City in 1999 from the local health department, a census track boundary map and population data from the US Census Bureau, and vegetation data from a United States Geological Survey (USGS) Landsat image [29]. Kulldorff's spatial scan statistic using a Poisson distribution model was used to detect clusters of West Nile. Once the clusters were detected, the authors used ANOVA to examine whether Normalized Difference Vegetation Index (NDVI) differed between clusters and non-clusters and used Logistic regression to determine if NDVI could identify census tracks within clusters that had cases of WNV. The authors concluded that mosquito and vegetation data, in conjunction with the spatial analysis might provide early evidence of human WNV risk [29].

### Analytic Linkage of Human and Animal Data Using Mathematical Modeling

Another analytic linkage method for animal and human data we found was mathematical modeling. Many researchers use dynamic stochastic models in which simulated data based on various distributions is represented as differential equations. For example, Li et al [36] used a probability model to examine the relationship between sentinel animals for transmission of arboviruses. As part of their work, the authors examined the relationship between various levels of vector bites per host per season and the mean number of human cases of an arbovirus [36]. Other calculated parameters in their model included "vector infection rate" ([36], pg. 450), "probability of an animal or human host becoming infected" ([36], pg. 450) and "the number of seroconverted samples on each sampling date" ([36], pg. 450). The authors concluded that human risk is "negatively related to the distance from the vector epicenter" ([36], pg. 450). These models can then be used as a means to predict risk of human infection given actual ecological data.

### Analytic Linkage of Human and Animal Data Using Risk Ratios

Several of the papers that used analytic linkage of animal and human data used direct calculation of risk ratios such as relative risk and odds ratios. These can often be calculated directly using contingency tables. The ratios are often used in active surveillance studies where the number of observations and variables collected is relatively small. For example Mannelli et al [37] calculated relative risk to determine the association between occupation and exposure to tick bites for Lyme Disease, while Gurtler et al [33] calculated the relative risk for the combined effect of T. cruzi in dogs and children and infected Triatoma infestans (insects). The authors performed a house-to-house survey and performed laboratory analysis on all household members, dogs, and Triatoma infestans. They determined that a causal association existed between the presence of infected dogs and infected insects [33].

### Analytic Linkage of Human and Animal Data Using Cross Correlation Functions

Cross-correlation function (CCF) has been used as a method to identify leading indicators of disease outbreaks [53]. The emphasis here is to examine the relationship between time series [53] to determine the lead-lag correlation. CCF have been widely used to examine various financial indices and the fluctuations in the stock market [54]. Two of the papers in the Canary Database that use analytic linkage used the cross-correlation function. In Niklasson et al [42], the authors used CCF to examine the relationship between human hantavirus infection and vole density. The authors concluded that in the Fall months, there was more of a concurrent relationship between vole density and human infection, whereas in the Spring, human infection was dependent on the density in the preceding Fall months [42]. In this Swedish epidemiological study, the authors utilized national incidence data from 1985–1992 and trapping of voles [42].

## Discussion

Examples of public health scenarios in which animal data can be used to inform decision making include:

1. Linkage of surveillance data streams through reporting of companion, livestock, or wildlife populations in order to gauge human risk.

2. Surveys of animal reservoirs to support a specific human outbreak investigation.

3. Surveys of animal reservoirs in the absence of a known human outbreak to gauge human risk and determine the need for public health and animal control measures.

4. Short-term surveys of unusual clusters of disease in animals that can lead to hypothesis about human risk.

5. Intentional sentinels (captive chickens, cows, etc.) followed in a cohort fashion to gauge increase or decrease in human risk.

6. Analysis and comparison of available human and animal isolates of an infectious agent to monitor genetic shifts in infectious agents.

Scenario 1 involves domestic or livestock animal medical cases by veterinarians or general public reporting of wildlife (such as dead birds). The animal health data in this scenario is reported to agencies such as the Departments of Agriculture, Wildlife, or Public Health. Many of these involve cases of suspected reportable animal diseases to the Department of Agriculture or public sightings of dead birds to the Department of Wildlife or Department of Health. An example is the use of dead crow sightings for West Nile Virus surveillance [20].

Scenario 2 involves outbreak investigation of human cases by obtaining health data on animals. The majority of disease surveillance in wildlife is short-term and often in response to known cases or spread of the disease [55]. This generally involves trapping, seroprevalence, and necropsy of wildlife in the vicinity of the human cases. It might also involve the testing of domestic animals in the belief of pet-owner transmission. These surveys are often initiated by governmental agencies such as the Department of Agriculture, Wildlife, or Public Health. An example is the investigation of anthrax in cattle and sheep on numerous Australian farms [56].

Scenario 3 is short or long-term seroprevalence surveys, typically of wildlife animals, to estimate the degree of infection of a specific zoonotic disease in animals. These surveys can be initiated by governmental agencies or also the result of independent research by university scientists. Trapping and testing rodents for plague in a country in order to estimate the levels of infection in wildlife is an example of this scenario [57].

Scenario 4 is similar to scenario 2, but is initiated by the discovery of unusual animal deaths, not human deaths.

Scenario 5 involves the use of specific sentinel animals (generally livestock) for long-term assessment of infection rates. An example is the use of sentinel chickens and horses for West Nile virus assessment as part of the ArboNET surveillance program [58].

Scenario 6 differs from the other scenarios because of its emphasis on laboratory techniques in the domain of molecular epidemiology. The intention is to examine similarities between strains of a pathogen occurring in animals compared to humans, thereby providing evidence as to whether or not species crossover of infection has occurred. An example is the development of phylogenetic trees to examine similarities between animal and human strains of Hantavirus [59].

In our analysis, descriptive linkage was the most common linkage category found. The use of animal data to quantitatively predict risk in human was limited. This lack of analytic linkage can impact the development of automated public health informatics systems to support surveillance. Analytic linkage is inherent in early warning surveillance systems for predicting risk in humans based on a range of variables (syndromes, animals, behavior, environment, etc.). For example, value has been shown in the use of dead crows and infected mosquitoes for predicting human risk of West Nile Virus [2,15,20,22]. Without this evidence, it is likely that much less funding would go into development of early warning WNV systems that link animal and human data. A lack of analytic linkage will produce a lack of scientific merit for using animals as sentinels for estimating human risk. This is a global problem as noted by Childs, that "as of 2006, there appears to be little scientific, social, or political consensus that animal-based surveillance for zoonoses merits investment in international infrastructure." pg. 423 [55]. The author does note that recent collaborations by the World Health Organization (WHO), Food and Agricultural Organization (FAO), and the World Organization for Animal Health (OIE), to develop GLEWS, an integrated early-warning system, might signal a change in the right direction [55].

In addition descriptive linkage is suitable for public health researchers looking at discrete events, but it can get overwhelming if there is too much data. The reliance on descriptive linkage puts a burden on public health users and also creates discrepancies in how different users interpret the results. For example, as more governmental money has gone into the epidemiology of Highly Pathogenic Avian Influenza (HPAI), many studies have been initiated and large amounts of data have been generated. However, the onus is on the researcher to analyze the descriptive data across all of these studies and draw conclusions without the aid of analytic methods for linking the findings. Interpretation of the data determines if public health control measures should be implemented. For zoonoses outbreaks, these measures can carry significant financial consequences such as the slaughtering of animals, and the cessation of trade.

We believe the three scenarios that tend to have long-term data collection (1, 3, and 5) benefit most from the use of analytic linkage between human and animal data. For example, scenario 1 is a long-term focus with passive data collection that relies on the motivation of veterinarians or the general public to report suspected cases. Scenario 1 is well suited for public health informatics systems that transfer, manage, and analyze longitudinal case data. The collection of cases over many years allows for analytic linkage of human and animal data through temporal and geographical comparisons. However, the lack of integrated informatics systems at animal and human agencies makes analysis of longitudinal health and animal data difficult. Animal and human agencies that do not use these systems to manage their case data make it difficult to integrate animal and human health data and conduct quantitative linkage. While it is encouraging that large numbers of papers have attempted to look at linking animal and human data, more emphases needs to be in quantitatively linking animal and human data. This will provide more scientific merit that animal data can be used as sentinels for predicting human risk, and hopefully promote the need for public health informatics surveillance systems to automatically integrate the animal and human data. In addition, the importance of the "One Medicine" [10] (or "One Health") collaboration needs to be emphasized across veterinary and human medicine through formal education, conferences and workshops, journal articles, and professional collaborations. An acceptance and belief of one medicine will heighten the recognition for using animal health data as a sentinel for determining risk in humans. It will also emphasize the importance of quantitative linkage between the two domains for producing empirical scientific evidence that will serve to strengthen this initiative and the development of automated animal-human informatics systems.

In our review of the Canary Database, we found different strategies for analytic linkage including: multivariate regression, direct calculation of risk ratios, spatial analysis including scan statistics, cross-correlation functions, and mathematical modeling. There are many other methods that were not found that might also be useful for analytic linkage of animal and human data. Other surveillance approaches include Bayesian Networks, rule-based analysis, and various time-series methods [60]. The authors here are not advocating one linkage approach over another. Each situation is different depending on the study goals, the prevalence of the disease in the area, additional variables collected, the number of observations, how many years of data were collected, and access to statistical and spatial software. It is possible that the key barrier to analytic linkage is not technological, but rather the lack of understanding of researchers of the importance of animal data as a 'sentinel' for human health. Our study highlighted that lack of animal-sentinel approaches in epidemiology and listed methods from the literature for performing these types of linkages. Researchers performing zoonotic surveillance should be aware of the value of animal-sentinel approaches for predicting human risk and consider analytic methods for linking animal and human data. Table 2 lists examples of Arboviruses such as West Nile Virus that use analytic linkage. As variables such as crow mortality and mosquito abundance have shown to be valuable for empirically predicting human risk, follow-up studies using additional data sources and analytic linkage efforts have been performed. As other research initiatives in public health provide empirical evidence for animal-sentinel surveillance, more scientific work that uses analytic methods to link this information will be performed. Finally, efforts such as the One Health Initiative [61], which focus on the collaboration of human and animal medicine, represent important collaborations to address this lack of recognition between the value of animal-human linkage. Going forward, qualitative work needs to be done in order to examine researchers' decisions in linkage strategies between animal and human surveillance data.

## Limitations

While the Canary Database is not an exhaustive compilation of all scientific studies relevant to issues of animal sentinels for human health risk, it does represent a systematic attempt to sample the published scientific literature from 1966 to 2007 to identify such studies. It therefore was a reasonable sample to use for this attempt to characterize the nature of epidemiological linkages between human and animal health data related to environmental exposure risks.

## Authors' contributions

MS conducted the literature review, developed the taxonomy, performed the analysis, and wrote the manuscript. LO conducted the literature review, developed the taxonomy, and reviewed the manuscript. PR developed the taxonomy and reviewed the manuscript. All authors read and approved the final manuscript.
